# Task-oriented telerehabilitation for upper limb functional recovery after stroke: a retrospective cohort study

**DOI:** 10.3389/fneur.2025.1611565

**Published:** 2025-08-21

**Authors:** Xiaojuan Hong, Huanghong Cha, Xiao Bao, Jinning Luo, Xiuling Li, Jinling Cheng, Zicai Liu

**Affiliations:** ^1^Department of Rehabilitation Medicine, Yuebei People's Hospital, Shaoguan, China; ^2^Department of Rehabilitation Medicine, Shaoguan First People's Hospital, Shaoguan, China

**Keywords:** stroke, telerehabilitation, task-oriented training, clinical trials, upper limb dysfunction

## Abstract

**Backgrounds:**

In clinical practice, many patients cannot undergo inpatient rehabilitation in hospitals for extended periods due to personal financial constraints, as well as China’s health insurance policy. They are often forced to terminate their rehabilitation training during the prime recovery phase. This makes tele-rehabilitation-based, home-based rehabilitation particularly important.

**Purpose:**

This retrospective cohort study aimed to compare the efficacy of tele-rehabilitation-based task-oriented training (TOT) versus face-to-face task-oriented training and conventional tele-neurofacilitation techniques.

**Methods:**

Patients who met the criteria were assigned to either the telerehabilitation group, the FTF group, or the Tele-Control group while receiving standardized rehabilitation treatment and education. Moreover, the Tele-Rehab group underwent tele-rehabilitation-based task-oriented training, the FTF group underwent face-to-face task-oriented training, and the Tele-Control Group underwent tele-rehabilitation-based conventional neurofacilitation techniques. The main evaluation indices were the Fugl-Meyer Assessment Upper Extremity Scale (FMA-UE), Wolf Motor Function Test (WMFT), and Action Research Arm Test (ARAT). Secondary outcome indicators were Instrumental Activities of Daily Living (IADL). All patients underwent 3 weeks of treatment.

**Results:**

In total, 79 participants completed the trial: Tele-rehab group (*n* = 23), FTF group (*n* = 28), and Tele-Control group (*n* = 28). Improvements in FMA-UE, WMFT, ARAT, and IADL were found in all three groups (*p*<0.05). The mean change in FMA-UE was 9.4 in the Tele-rehab group, 6.4 in the FTF group, and 6.7 in the Tele-control group. The mean difference between the Tele-Rehab and FTF groups was 3.0, and the mean difference between the Tele-Rehab and Tele-Control groups was 2.7, with the upper limit of the 95% confidence interval not exceeding the margin of non-inferiority. Non-inferiority was demonstrated, as the 95% CI did not cross the margin in FMA-UE difference scores before and after the intervention in the Tele-rehab group compared with the FTF group (*p* > 0.05), nor in the FTF group compared with the Tele-Control group before and after the intervention (*p* > 0.05). The 95% CI for FMA-UE improvement between Tele-rehab TOT and face-to-face TOT was [−0.81, 7.39], not exceeding the non-inferiority margin of 12.4.

**Conclusion:**

Task-oriented training and remote traditional neurofacilitation techniques for tele-rehabilitation of stroke patients can enhance upper limb motor function and improve quality of daily life with comparable efficacy to face-to-face task-oriented training. Therefore, telerehabilitation is a method that is not inferior to conventional rehabilitation and deserves to be used and promoted in homebound patients.

## 1 Introduction

Stroke is a common cerebrovascular disease, comprising ischemic stroke (80%) and hemorrhagic stroke (20%) (1). Approximately 60–80% of stroke survivors experience motor dysfunction (2, 3), with upper limb impairment being particularly prevalent (4). This impairment not only hinders daily activities and reduces quality of life (5–7) but also imposes significant economic burdens on families and society (8). Stroke patients in the early stage of stroke are usually in the hospital under the guidance of a rehabilitation therapist, and since most stroke patients still have functional impairment after discharge from the hospital (9), they need to undergo continuous rehabilitation training. However, routine rehabilitation training in hospitals is difficult for some patients, which is often related to treatment resources (transport, professionals (10), funding sources), etc., which will also affect subsequent recovery. Therefore, exploring rational training methods and effective training modalities is necessary (11, 12).

Tele-rehabilitation (TR) refers to the provision of rehabilitation services through the use of telecommunication devices such as mobile phones, computers, and tablets in combination with information and modern communication technologies (13). TR is a promising model that features remotely managed treatment, effectively expanding service accessibility and treatment modalities, which compensates for the lack of rehabilitation treatments received by the patient due to the high cost, the scarcity of professional treatment staff, and the lack of transport (14). In addition, there have been several studies pointing out the good efficacy of, such as in 2018, Huidi Tchero et al. showed that TR is comparable to conventional care in terms of improving patients’ quality of life and other aspects, as well as lower costs (15). However, there are some limitations due to the lack of close guidance from professionals, such as a recent Meta-analysis indicated that the efficacy of TR was comparable to that of conventional rehabilitation, but patients undergoing TR tended to have poorer adherence due to differences in training schedules and conditions, etc. (13). Meanwhile, another meta-analysis showed low evidence that remote self-rehabilitation efficacy is comparable to face-to-face conventional efficacy (16).

Task-oriented training (TOT) is a neuroscience-based intervention (17) that has been used in the rehabilitation of stroke patients (18), which focuses on the repetition of multiple explicit active training sessions (19). Relevant literature suggests that TOT is effective in improving motor and functional recovery by incorporating principles of neuroplasticity (20, 21). Similarly, Annick et al. stated in a systematic evaluation that task-oriented training consists of 15 components, some of which improve patients’ ability to remember learned motor performance (22). However, most TOT is currently performed by professionals in a defined clinic or research setting (23), which limits its applicability to some extent. Neurofacilitation techniques are commonly used for CNS injuries, including the Bobath technique, the Brunnstrom technique, and the PNF technique. Neurofacilitation techniques emphasize the stimulation and modulation of neurons in the motor pathway through different methods, which have a wide range of clinical applicability and a high degree of patient cooperation.

Current applications of TR in stroke rehabilitation remain debated. While studies confirm its non-inferiority to conventional care (15), recent systematic reviews note significant adherence and technical standardization challenges (13). In rural China, <30% of patients access TR due to uneven healthcare resources and digital disparities (24), underscoring the need for context-specific protocol optimization. Crucially, no studies have compared TR-delivered task-oriented training (TOT) against neurofacilitation techniques using non-inferiority designs—a gap this study addresses.

To further compare the efficacy of TR combined with task-oriented training and Neurofacilitation techniques, this study, therefore, used a non-inferiority design to compare the effects of task-oriented training with TR, face-to-face task-oriented training, and conventional neurofacilitation techniques treatments on upper limb dysfunction after stroke.

## 2 Methods

### 2.1 Study design

This non-inferiority trial utilized a retrospective cohort design based on historical data from patients who received different interventions. Eighty-nine stroke patients with upper limb motor dysfunction who attended the Department of Rehabilitation Medicine of Yuebei People’s Hospital from January 2021 to June 2024 were selected. Depending on the type of intervention, the researchers assigned patients to 3 groups (Grouping based on historical data). Each patient had to receive standardized rehabilitation treatment and education simultaneously. The Ethics Committee of Yuebei People’s Hospital approved the study under No. KY-2022-101. Considering the type of retrospective study, the right to exemption from informed consent was obtained after ethics committee approval. The ethics committee granted a waiver of informed consent under the condition that all data were anonymized and retrospectively analyzed without compromising patient privacy (e.g., deletion of name, medical record number, etc). Assignments between groups are grouped according to the historical events that have been accomplished.

### 2.2 Eligible criteria

*Inclusion criteria were*: (1) first onset; (2) all patients with ischaemic or hemorrhagic stroke meeting the diagnostic criteria established by the Fourth National Academic Conference on Cerebrovascular Disease and confirmed by MRI or CT; (3) functional motor disorders of the upper limb and hand on the hemiplegic side; (4) stable vital signs, and duration of the disease between 3 and 24 months; (5) age between 18 and 75 years old; (6) upper limb Functional Brunnstorm stage 1 to 4; (7) clear consciousness, no serious cognitive impairment, no auditory or visual impairment; (8) Walking ability: Functional Ambulation Category≥3. (9) Ashworth spasticity scores: I^+^- II (1.5–2).

*Exclusion criteria were*: (1) aphasia, apraxia, lateral neglect; (2) cognitive dysfunction: mini-mental state examination (MMSE) score <24 points (secondary school level or above), illiteracy <17 points; (3) severe spasticity of the upper limbs, Ashworth > 3; (4) previous other brain diseases or history of brain surgery; (5) other causes of severe spasticity of the upper limbs or upper limb dysfunction; (6) unstable condition or vital organ failure, malignant tumor; (7) inability to cooperate with the completion of the MRI examination, etc.; (8) women during pregnancy.

### 2.3 Interventions

The Tele-Rehab group underwent task-oriented training guidance as the treatment method of TR therapy, the FTF group receives face-to-face task-oriented training and rehabilitation therapy, and the Tele-Control group receives standardized self-guided neurofacilitation exercises via a pre-recorded video platform, with weekly check-ins to ensure protocol adherence (see Table 1). The duration of each treatment in each group was 1 h per day, 6 days per week for 3 weeks. Total dose: 1 h/day, 6 days/week × 3 weeks (18 h total).

**Table 1:** Intervention protocols.

Group	Content	Delivery mode	Intensity/Parameters	Progression
Tele-Rehab	Task-oriented training (e.g., cup grasping, drawer pushing, turning keys)	Synchronous video (WeChat); real-time correction; household items used	60 min/session; 6 sessions/week; 3 weeks	Task complexity ↑ weekly based on performance
FTF	Identical TOT tasks with standardized equipment (pegboards, weight disks)	In-clinic 1:1 sessions; standardized equipment	Identical to Tele-Rehab	Identical progression
Tele-Control	Neurofacilitation (Brunnstrom staging: passive ROM → active-assisted exercises)	Asynchronous videos; weekly check-ins; caregiver supervision	60 min/session; 6 sessions/week	Resistance ↑ weekly based on performance

### 2.4 Study procedures

The Tele-Rehab group requires the patients to carry out targeted, targeted, and systematic upper limb task-oriented training at home under the remote guidance of the therapist. The FTF group was treated by a therapist in an occupational therapist for face-to-face, task-oriented training. In the Tele-Control group, patients were asked to carry out upper limb rehabilitation therapy at home on their own. Quality control: Dual camera angles (wide + close-up), therapist error screening every 15 min, daily video logs, therapist video audits (exercises with >30% deviation triggered in-person reassessment).

### 2.5 Outcome measures

After randomized grouping, patients were evaluated and followed up by the therapist at baseline (before treatment), after treatment, or before discharge.

#### 2.5.1 Main evaluation indicators

##### 2.5.1.1 Fugl-Meyer Assessment Upper Extremity Scale (FMA-UE)

The FMA-UE assesses the recovery of the shoulder, forearm, elbow, wrist, and hand. The specific assessment consists of 33 items, each of which is scored on three levels: 2 for full conduction, 1 for partial conduction, and 0 for no conduction. With a maximum score of 66 and a minimum of 0, the scale has high reliability and validity, with higher final scores indicating better function (25, 26).

##### 2.5.1.2 Wolf Motor Function Test (WMFT)

The WMFT-FAS assesses upper limb motor function and consists of 15 items ranging from simple forearm touching to a table to complex card flipping, etc. A score of 0 indicates that the affected side cannot be used, and a score of 5 indicates full ability to perform the task. The scale has high reliability and validity, with higher final scores indicating better function (27).

##### 2.5.1.3 Action Research Arm Test (ARAT)

The ARAT assesses the activity and participation of the upper extremity. The specific assessment consists of 19 items, each of which consists of four components: grasping, grip strength, pinching, and gross motor. Each item is scored from 0 to 3, with 0 indicating no movement and 3 indicating full completion. The maximum score is 57. The scale has high reliability and validity, with higher final scores indicating better functioning.

#### 2.5.2 Secondary outcome indicators

The Internal Activities of Daily Living (IADL) assesses the patient’s functional independence. The specific questionnaire consists of eight components: responsibility for taking medication, ability to handle finances, preparing food, using the telephone, shopping, doing housework, washing personal clothes, and using transport. The scale has high reliability and validity, with higher total scores indicating better independence.

### 2.6 Statistical analysis

The analysis included measures such as median (IQR) for continuous variables and frequencies and percentages for categorical variables. Group comparisons for continuous variables were performed using the Wilcoxon rank-sum test or the Kruskal-Wallis test. For comparison between groups of categorical data, we used the Fisher exact test for expected frequencies <5; otherwise, we used the Chi-squared test.

The efficacy evaluation is mainly based on the concept of double difference. Analysis of variance (ANOVA) was used to compare the change (gain) in each outcome indicator before and after treatment between groups. Home-based TR training was considered non-inferior to face-to-face task-oriented training during hospitalization if the upper limit of the 95% confidence interval (CI) for the difference in FMA-UE gain before and after the two treatments was not greater than the noninferiority margin. The non-inferiority margin was set at 12.4, which has been reported to be the minimum clinically important difference (MCID) for FMA-UE in stroke patients (28, 29). Although not directly validated in our cohort, this threshold aligns with clinical relevance, and prior studies were also referenced (28, 29). Priori sample size calculation was performed based on the non-inferiority margin (MCID = 12.4), with an assumed standard deviation of 8.8 (from pilot data), alpha = 0.05, and power = 80%, yielding a required sample size of 25 per group. Post-hoc power analysis confirmed 78.5% power - above the 75% minimum recommended for non-inferiority trials (25, 26), the final sample size (*n* = 79) met this requirement.

Subgroup analyses were performed to examine differences across subgroups (gender, stroke type, and lesion side) and whether there is any difference in efficacy, the least squares mean and its confidence interval based on the ANOVA model and the *p*-value showing the interaction can help to determine the difference between different groups, so we selected the FMA as an outcome indicator to draw the forest plot for subgroup analysis. In our study, all statistical analyses were performed using the R software (version 4.2.2).

## 3 Results

**Figure 1:**
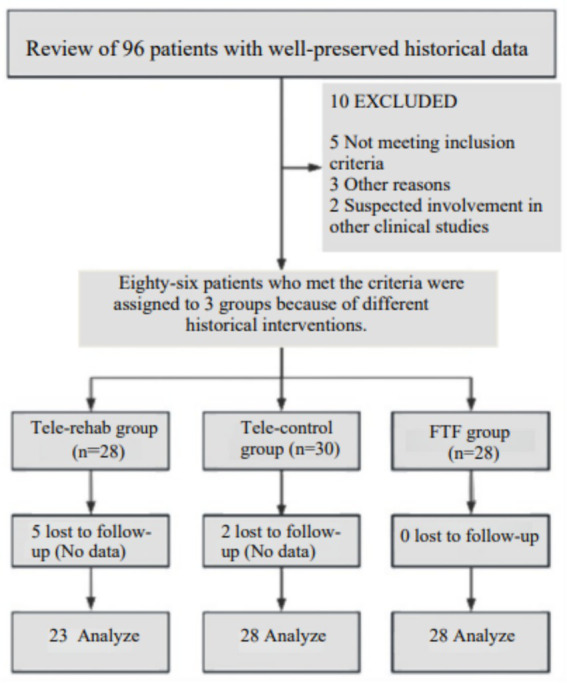
Patients recruitment flowchart.

A total of 96 stroke patients were recruited, 10 patients were excluded for various reasons, 86 people were categorized into three groups because of the different types of intervention, of which 7 without data and withdrew from the study. A total of 79 subjects completed the trial (see Figure 1 for the inclusion flowchart), and no adverse reactions were reported. General information, including mean age, MMSE scores, effective duration of intervention, gender, stroke type, and site of injury, was statistically analyzed across the three groups, and the differences were not statistically significant (*p* > 0.05). There was a significant difference in the disease duration among the three groups (*p*<0.001), with the Tele-Rehab group having the longest disease duration, followed by the Tele-Control group, and the shortest being the FTF group, we used ANCOVA to correct for differences in disease duration. The detailed results are shown in Table 2.

**Table 2:** Patient demographics and baseline characteristics.

Characteristic	Group			*p*-value
	Tele-Control, *N* = 28^1^	FTF, *N* = 28^1^	Tele-Rehab, *N* = 23^1^	
Age	57 (47, 66)	60 (53, 63)	60 (50, 63)	0.646^2^
MMSE	27.5 (25.5, 30.0)	27.5 (23.8, 30.0)	26.0 (24.5, 30.0)	0.642^2^
Disease duration (days)	47 (41, 62)	29 (21, 44)	70 (48, 140)	<0.001^2^
Treatment time	32.0 (28.0, 32.3)	29.5 (25.8, 31.0)	30.0 (29.0, 31.0)	0.112^2^
Sex				0.733^3^
Female	8 (28.6%)	10 (35.7%)	6 (26.1%)	
Male	20 (71.4%)	18 (64.3%)	17 (73.9%)	
Stroke type				0.113^3^
Hemorrhage	10 (35.7%)	8 (28.6%)	13 (56.5%)	
Infarction	18 (64.3%)	20 (71.4%)	10 (43.5%)	
Lesion side				0.972^3^
Left	15 (53.6%)	15 (53.6%)	13 (56.5%)	
Right	13 (46.4%)	13 (46.4%)	10 (43.5%)	
FAC				0.809^3^
FAC-3	9 (32.1%)	7 (25.0%)	5 (21.7%)	
FAC-4	13 (46.4%)	17 (60.7%)	13 (56.5%)	
FAC-5	6 (21.4%)	4 (14.3%)	5 (21.7%)	
Ashworth spasticity				0.096^3^
I+	10 (35.7%)	12 (42.9%)	15 (65.2%)	
II	18 (64.3%)	16 (57.1%)	8 (34.8%)	

^1^ Median (IQR); *n* (%); ^2^ Kruskal-Wallis rank sum test; ^3^ Pearson's Chi-squared test.

FAC, Functional Ambulation Category.

### 3.1 Major outcomes

Before treatment, the scores of the three groups of patients were compared; the differences were not statistically significant (*p* > 0.05) and were comparable. After treatment, we found that the scores of the Tele-Rehab group, the FTF group, and the Tele-Control group were significantly improved compared with those of the pre-treatment group (*p* > 0.05). Detailed results are shown in Table 3. The non-inferiority results showed (see Table 4) that the FMA-UE scores of the Tele-Rehab group were non-inferior to those of the FTF group and the Tele-Control group after the treatment, and the difference between the pre-and post-treatment values was also non-inferior to those of the other two groups. The 95% CI for the difference in change between FMA-UE treatment in the test and FTF groups did not exceed a non-inferiority margin of 12.4, indicating that treatment in the test group was non-inferior to that in the FTF group (mean [SD] of 9.4 (8.80) in the test group and 6.4 (7.52) in the FTF group; mean difference between groups: 3.29; 95% confidence interval: (−0.81, 7.39)). The 95% CI for the difference in change between the Tele-rehab and Tele-Control groups, and the FTF and Tele-Control groups also did not exceed a non-inferiority margin of 12.4. The Tele-Rehab group showed a numerically greater improvement in FMA-UE compared to both the FTF and Tele-Control groups, although the difference did not reach statistical superiority (*p* > 0.05).

**Table 3:** Comparison between groups before and after treatment.

Characteristic		Group			*p*-value
		Tele-Control, *N* = 28^1^	FTF, *N* = 28^1^	Tele-Rehab, *N* = 23^1^	
FMA	Baseline	16 (9, 38)	20 (6, 37)	29 (11, 45)	0.683^2^
	Post-treatment	28 (13, 50)	31 (12, 43)	35 (21, 55)	0.499^2^
WOLF	Baseline	7 (5, 23)	12 (2, 22)	20 (7, 30)	0.520^2^
	Post-treatment	17 (11, 35)	16 (7, 36)	26 (14, 39)	0.520^2^
ARAT	Baseline	3 (3, 12)	4 (3, 11)	3 (3, 13)	0.820^2^
	Post-treatment	8 (3, 26)	6 (3, 20)	6 (4, 25)	0.679^2^
IADL	Baseline	9.0 (6.0, 13.3)	8.0 (6.0, 11.0)	10.0 (6.0, 11.0)	0.380^2^
	Post-treatment	10.5 (8.0, 13.3)	9.0 (7.0, 11.3)	12.0 (8.5, 14.0)	0.103^2^

FMA, Fugl-Meyer Assessment Upper Extremity Scale; WOLF, Wolf Motor Function Test; ARAT, Action Research Arm Test; IADL, Instrumental Activities of Daily Living; Tele-Control, Tele-Control; FTF, Face to Face; Tele-Rehab, Tele-rehabilitation.

**Table 4:** Effectiveness analysis.

Group	Baseline		Post-treatment-FMA		Change from baseline		
	*N*	Mean (SD)	N	Mean (SD)	*N*	Mean (SD)	LS Mean (95% CI) ^a^
FTF	28	24.9 (19.48)	28	31.2 (19.58)	28	6.4 (7.52)	6.31 (3.52, 9.10)
Tele-Control	28	25.1 (20.49)	28	31.8 (20.18)	28	6.7 (6.28)	6.62 (3.83, 9.41)
Tele-Rehab	23	28.1 (18.62)	23	37.6 (18.67)	23	9.4 (8.80)	9.61 (6.52, 12.69)

Pairwise comparison	Difference in LS Mean (95% CI) ^a^	*p*-value
(Tele-Control) - FTF	0.31 (−3.58, 4.19)	0.987
(Tele-Rehab) - FTF	3.29 (−0.81, 7.39)	0.263
(Tele-Rehab) - (Tele-Control)	2.99 (−1.11, 7.08)	0.332

^a^ Based on an ANCOVA model after adjusting baseline FMA. ANCOVA, Analysis of Covariance; CI, Confidence Interval; LS, Least Squares; SD, Standard Deviation.

For the primary outcome (FMA-UE), the 95% CI of the between-group difference [−0.81, 7.39] did not cross the non-inferiority margin (12.4). The upper limit (7.39) was 5.01 points below the MCID - exceeding FMA-UE’s typical standard error of measurement (SEM = 3.2 points (24)). Sensitivity analysis using bias-corrected bootstrapping yielded consistent results (95% CI: −0.75 to 7.31).

### 3.2 Secondary outcomes

The difference in IADL scores among the three groups before treatment was not statistically significant (*p* > 0.05) and was comparable. For secondary outcomes (WMFT, ARAT, IADL): Group differences had narrower CIs (e.g., WMFTΔ: 95% CI [−1.22, 5.18]) due to lower variability (SD < 6.0). No significant interactions were found in subgroup analyses (all *p* > 0.10), suggesting consistent effects despite group size differences. Demonstrates that the primary concern (wide CI) was isolated to FMA-UE and did not affect other outcomes, highlights internal consistency of results.

### 3.3 Subgroup analyses

**Figure 2:**
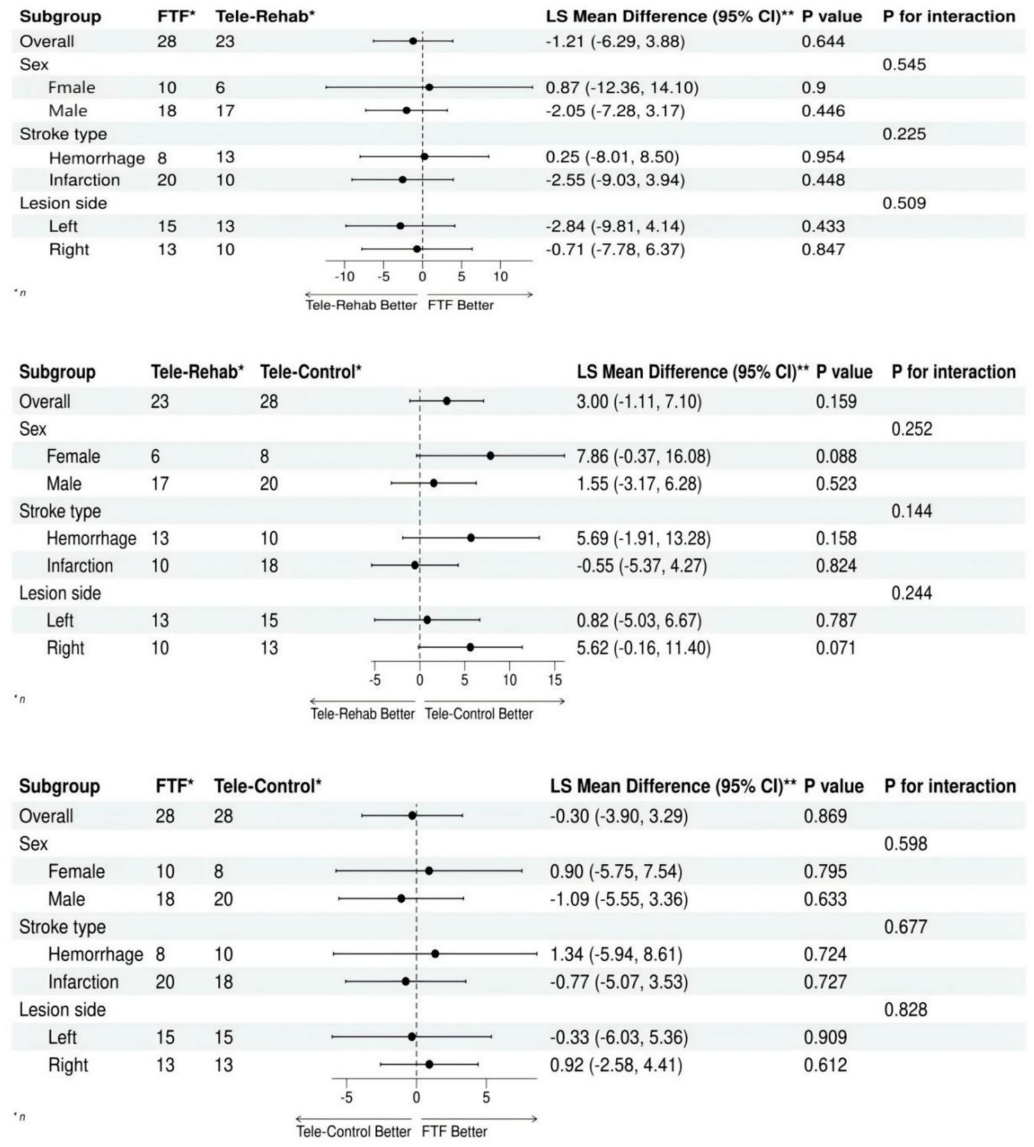
Forest plot of subgroup analysis for FMA-UE.

Subgroup analyses of FMA, the primary outcome indicator, were conducted at the end of treatment to assess the impact of gender, stroke type, and stroke site on outcomes. It was found that efficacy was not affected by gender, stroke type and stroke site. Specifically, see Figure 2.

## 4 Discussion

The results of this study found that the scores of all groups were higher after the treatment than before the treatment, in which the difference between the pre and post-test scores of the Tele-Rehab group was not inferior to that of the conventional and Tele-Control groups. The above results suggest that TR or face-to-face combined with task-oriented training or tele-conventional neurological easing techniques can improve the upper limb motor function and daily living ability of stroke patients.

Stroke often affects the control ability of some areas of the brain due to the death of brain cells (30), which leads to some functional impairment after a period of rehabilitation (31), upper limb motor dysfunction is often more serious than the lower limb (32), and upper limb motor dysfunction has a great impact on the quality of life (33, 34), so there is an urgent need for highly operable and effective training programs (35). Recent studies have further demonstrated that changes in cortical-muscular coupling in stroke patients are significantly associated with clinical function scores (e.g., FMA-UE), which provides physiological evidence that task-oriented training improves motor function through neuroplasticity mechanisms (36).

Our TR-TOT adherence rate (92%) exceeded the 78% reported in Tchero et al.’s study (15), likely due to hybrid synchronous-asynchronous supervision (real-time correction + weekly audits). The FMA-UE improvement (∆9.4) aligns with Cramer et al.’s home-based telerehabilitation trial (∆8.7) (10), but contrasts with Laver et al.’s finding of superior clinic-based outcomes (9). This discrepancy may stem from our ecologically valid tasks (e.g., cup grasping) versus standardized equipment training. Notably, TR neurofacilitation (∆6.7) showed comparable efficacy to face-to-face TOT (∆6.4), supporting Chen et al.’s (25) assertion. Self-guided protocols suffice for structured techniques like Brunnstrom. From the perspective of training modality, we found that TR combined with task-oriented training could improve patients’ ability to perform daily living activities, and the Mean (SD) of the difference between pre-and post-FMA-UE scores, which was not inferior to task-oriented training performed face-to-face. Statistically corrected results support non-inferiority despite potential confounders (e.g., environmental differences). It cannot be denied that TR, as a means of providing online services for rehabilitation (27), is non-inferior to face-to-face rehabilitation training, and the results are independent of gender, type of lesion, and lesion location. In addition, TR has the characteristics of low cost and is not affected by geography (24). Consequently, with the popularity of the technology and the individualization of the training intensity, etc., it may be widely applied to stroke patients who require long-term rehabilitation training.

From the perspective of training methods, we found that both remote task-oriented training and conventional neural facilitation techniques can improve upper limb function in stroke patients, and the efficacy of both is comparable. Task-oriented training emphasizes a task-oriented strategy, which aims to train patients toward a certain goal through targeted and repetitive training (37) and to provide certain incentives (38), as well as to develop their ability to adapt to different environments (22, 39). Neurofacilitation techniques are based on physiological and developmental theories to restore motor function, emphasizing the therapist’s skills and the patient’s cooperation usually without incentives. The results of this study show that task-oriented training and conventional neuro-easy techniques are equally effective in restoring upper limb function in stroke patients, suggesting that patients can choose the appropriate method according to their needs and acceptability. The results of this study may be related to the differences arising from the patients’ degree of self-management and training standardization. Finally, most of the patients only trained for about 1 month, after which there was no follow-up, which could be followed up to observe the long-term efficacy of the different groups. TR-TOT’s non-inferiority may stem from: (1) Neuroplasticity: Home-environment training promotes skill generalization (40); (2) Self-efficacy: Patient autonomy enhances engagement (41, 42); (3) It’s also a dual cognitive + motor training (43), essentially a clinical application of ecological validity, − embedding rehabilitation scenarios into real-life environments and bridging the gap between traditional training and life practice.

This study has several limitations. First, the relatively small sample size (*n* = 79) may restrict the generalizability of findings and reduce statistical power to detect subtle differences between groups. Second, the non-inferiority margin (12.4 points on FMA-UE) was adopted from prior literature but lacked direct validation in our population, potentially affecting interpretation. The smaller sample in the Tele-Rehab group (*n* = 23) reduced precision for FMA-UE comparisons, as reflected in the wider confidence interval (−0.81 to 7.39). While statistically adequate for non-inferiority conclusions (power = 78.5%), future trials should recruit ≥35 participants per group to achieve 90% power and narrower CIs. Third, the 3-week intervention period and absence of long-term follow-up limit conclusions about sustained treatment effects. Fourth, while randomization was implemented, baseline disease duration differed significantly across groups (*p* < 0.001), introducing potential confounding. Additionally, TR protocols relied on patient self-reporting for adherence monitoring, and environmental factors (e.g., home setup quality) were not systematically controlled. Although disease duration differed significantly across groups, we adjusted for this variable in the ANCOVA model to minimize potential confounding. Finally, the single-center design and specific cultural context of China’s healthcare system may limit cross-regional applicability. The greater numerical improvement suggests that future studies need to further validate its potential benefits. Future research could incorporate emerging temporal-guided adaptive graph learning techniques (44) to more accurately identify patients’ coordinated movement patterns to optimize personalized tele-rehabilitation programs. There is also a trend toward combining neuromodulation techniques (45, 46) or home virtual reality (47), Constraint-induced movement therapy (48), and so on.

The efficacy of TR combined with task-oriented training is no less than that of face-to-face task-oriented training, and also the effectiveness of task-oriented training is comparable to that of neural efficiency training, with significant improvement in both upper limb dysfunction remaining in post-stroke patients and in reduced ability to perform activities of daily living.

## 5 Conclusion

This study has demonstrated that TR combined with task-oriented training is feasible and non-inferior to for upper limb dysfunction in stroke patients and that both task-oriented training and neural facilitation techniques are effective in promoting upper limb functional recovery in stroke patients. Many studies have indicated that a certain amount of dysfunction may still exist even months after stroke. If the TR platform can be improved and optimized in the future, this will be a great benefit to society and families.

## Data Availability

The original contributions presented in the study are included in the article/supplementary material, further inquiries can be directed to the corresponding authors.
